# Rab11a Is Overexpressed in Gastric Cancer and Regulates FAK/AKT Signaling

**DOI:** 10.1155/2020/3494396

**Published:** 2020-10-31

**Authors:** Jiang Du, Lin Fu, Jie Hao, Xiumin Lin, Qianze Dong

**Affiliations:** ^1^Department of Pathology, College of Basic Medical Science, China Medical University, The First Affiliated Hospital of China Medical University, Shenyang, China; ^2^University of Central Florida College of Medicine, Burnett School of Biomedical Sciences, Orlando, FL, USA

## Abstract

Dysregulation of Rab11a has been implicated in the progression of several cancers. However, there have been no such studies for human gastric cancers. In the current study, we examined Rab11a protein expression and found it was upregulated in 49 of 108 gastric cancer tissues and correlated with local invasion, nodal metastasis, and advanced stage. Rab11a protein was higher in gastric cancer cell lines than normal gastric cell line. We transfected Rab11a plasmid and siRNA in both MGC803 and AGS cell lines. Rab11a overexpression increased the cell growth rate, colony numbers, and invasion ability in both MGC803 and AGS cell lines. Downregulation of Rab11a using siRNA decreased the cell proliferation rate, colony numbers, and inhibited invasion. Rab11a overexpression also conferred cisplatin resistance. Annexin V/PI staining showed that Rab11a overexpression suppressed cisplatin-induced apoptosis, while Rab11a depletion promoted cell apoptosis. We also showed that Rab11a overexpression maintained mitochondrial membrane potential. Western blot analysis revealed that Rab11a increased protein expression of MMP2, cyclin D1, Bcl-2, p-FAK, and p-AKT, while Rab11a depletion showed the opposite effects. Blockage of FAK using inhibitor downregulated Bcl-2, cyclin D1, MMP2, and p-AKT expression and abolished the effects of Rab11a on these proteins. In summary, our data demonstrated that Rab11a is upregulated in human gastric cancers. Rab11a facilitated cell proliferation and invasion, as well as cisplatin sensitivity and mitochondrial membrane potential, possibly via the FAK/AKT signaling pathway.

## 1. Introduction

Gastric cancer is a common malignancy worldwide and it is one of the leading causes of cancer-related death. The prognosis of gastric remains poor during the past decades, especially for patients with advanced stage cancers [1]. Despite surgery and novel chemotherapeutic drugs has improved survival, the development of chemoresistance is one of the most important obstacles during treatment [2]. So, it is important to identify effective targets involved in progression and chemoresistance of gastric cancers.

Rab11a belongs to the Rab family proteins which control Rac activity [3]. Rab11a was identified as a protein involved in cell-cell communication during collective movement [4]. Rab11a is associated with endosomes and contributes to spindle pole assembly and function [5]. Recently Rab11a dysregulation has been implicated in several cancers. Rab11a is upregulated, regulates in colorectal carcinoma, and inhibits E-cadherin expression, which induces cell transformation [6]. Rab11a also activates Wnt/*β*-catenin signaling to enhance cancer progression in pancreatic cancers [7]. Rab11a is overexpressed in lung cancer and promotes cancer proliferation through regulation of Hippo signaling [8]. It was reported that Rab11a contributed to cancer growth and invasion and was a target of miR-320a in breast cancer [9]. These studies indicate Rab11a as a potential oncoprotein during cancer progression. However, its the clinical significance and biological roles in human gastric cancers remain unknown.

To address these questions, we evaluated Rab11a protein in gastric cancer tissues and analyzed its clinical significance. We also examined whether Rab11a could influence the biological behavior and investigated the possible mechanism.

## 2. Materials and Methods

### 2.1. Specimens and Ethics Statement

This study protocol was approved by the Institutional Reviewer Board of China Medical University. Gastric cancer specimens were obtained from patients diagnosed with gastric cancer patients between 2010 and 2015. Informed consent was provided by the patients. Clinical data including histopathological diagnosis and tumor grade were extracted from medical records. The histological diagnosis was evaluated for sections stained with hematoxylin and eosin according to the World Health Organization (WHO) classification guidelines. 10 cases of fresh tumor tissues with corresponding normal tissue were stored at −80°C after resection for protein.

### 2.2. Immunohistochemistry

Tissue paraffin sections (5 *μ*m) were deparaffinized using xylene and treated with graded alcohol (100%; 90%; 80%; 70%, 2 minutes each). Antigen retrieval was performed using citrate buffer (pH 6.0). Sections were then incubated with normal goat serum. Then sections were treated with Rab11a antibody (1 : 300 dilution, Proteintech, USA) overnight at 4°C. Immunohistochemical staining was performed using the Elivision plus kit (MaiXin, Fuzhou, China). Staining was developed with DAB plus kit (MaiXin, Fuzhou, China).

The slides were evaluated according to previous report [8]. Cytoplasmic localization was regarded as positive staining. Intensity was scored as 0 (no/weak staining), 1 (moderate staining), and 2 (strong staining). Score of staining percentage was classified as 1 : 1%–25%, 2 : 26%–50%, 3 : 51%–75%, and 4 : 76%–100%. Intensity and percentage scores were multiplied to the final score. Rab11a was considered low expression when the score was <4 and high expression when score was ≥4.

### 2.3. Cell Culture and Transfection

Normal cell line GES-1 and gastric cancer cell lines BGC-823, MGC803, AGS, HGC-27, and NCI–N87 were obtained from Shanghai cell bank of Chinese Academy of Sciences (Shanghai, China). These cells were maintained in PRMI-1640 with 10% fetal bovine serum (FBS) (Invitrogen).

Empty plasmid and pCMV6-Rab11a were obtained from Origene (Origene, USA) and transfected into cells using Lipofectamine 3000 (Invitrogen, USA). SiGENOME siRNA for Rab11a (Dharmacon, USA) was used for knockdown experiment using Dharmafect1 reagent (Dharmacon, USA).

### 2.4. Western Blotting

Protein samples were separated by SAS-PAGE and then transferred to a PVDF membrane which incubated with primary antibodies against Rab11a (1 : 1000; Proteintech), cyclin D1, MMP2, Bcl-2, p-AKT, p-FAK, FAK, AKT (1 : 1000, Cell Signaling, USA), and GAPDH (1 : 3000; Cell Signaling, USA) overnight. After incubation with secondary antibodies (1 : 2000, Santa and Cruz, USA). The western blot bands were visualized using ECL HRP substrate and recorded with DNR Bio-Imager.

### 2.5. Quantitative Real-Time PCR (SYBR Green Method)

RNA was extracted using RNAiso reagent from TaKaRa (Dalian, China). Reverse transcription was performed using TaKaRa RT kit (Dalian, China). Real-time PCR was carried out using SYBRGreen Mastermix (TaKaRa, Dalian, China) with ABI7500 PCR System (ABI, USA). *β*-Actin was used as endogenous control.

### 2.6. MTT and Colony Formation Assays

For MTT assay, 3000 cells were seeded in a 96-well plate 24 h after transfection. Then 20 *μ*l MTT (thiazolyl blue) solution was added to the wells. After 4 hours of incubation, the medium was discarded and the remaining formazan was dissolved using 150 *μ*l of DMSO. The plate was scanned using a plate reader.

For colony formation assay, 24 h after transfection, 1000 cells were seeded in 6 cm dishes. These cells were cultured for 14 days then these culture dished were stained with Giemsa.

### 2.7. Matrigel Invasion Assay

Matrigel invasion assay was carried out using Transwell chamber coated with 20 *µ*l Matrigel from BD bioscience. Cells were placed in the upper chamber with serum free medium. The lower chamber was placed with medium with FBS. After 24 h of incubation, cells invading through the membrane were fixed and hematoxylin staining was performed.

### 2.8. Apoptosis and Mitochondrial Membrane Potential

To determine the apoptosis percentage, Annexin V/PI staining kit (BD bioscience) was used for cell staining. Then cells with Annexin V/PI staining were analyzed with flow cytometer.

For detection of mitochondrial membrane potential (Δ*ψ*m), JC-1 dye (Cell Signaling Technology) was used to stain cells for 30 minutes. After that cells were washed and analyzed using flow cytometer.

### 2.9. Statistical Analysis

SPSS version 16.0 (SPSS, Chicago, IL, USA) was used for statistical analyses. The correlations between Rab11a levels and clinicopathological factors were analyzed using *X*2 tests. Student's *t*-test was used to compare data obtained from biological experiments. *P* < 0.05 was considered as statistical significance.

## 3. Results

### 3.1. Rab11a Protein Expression Is Upregulated in Gastric Cancers

**Figure 1:**
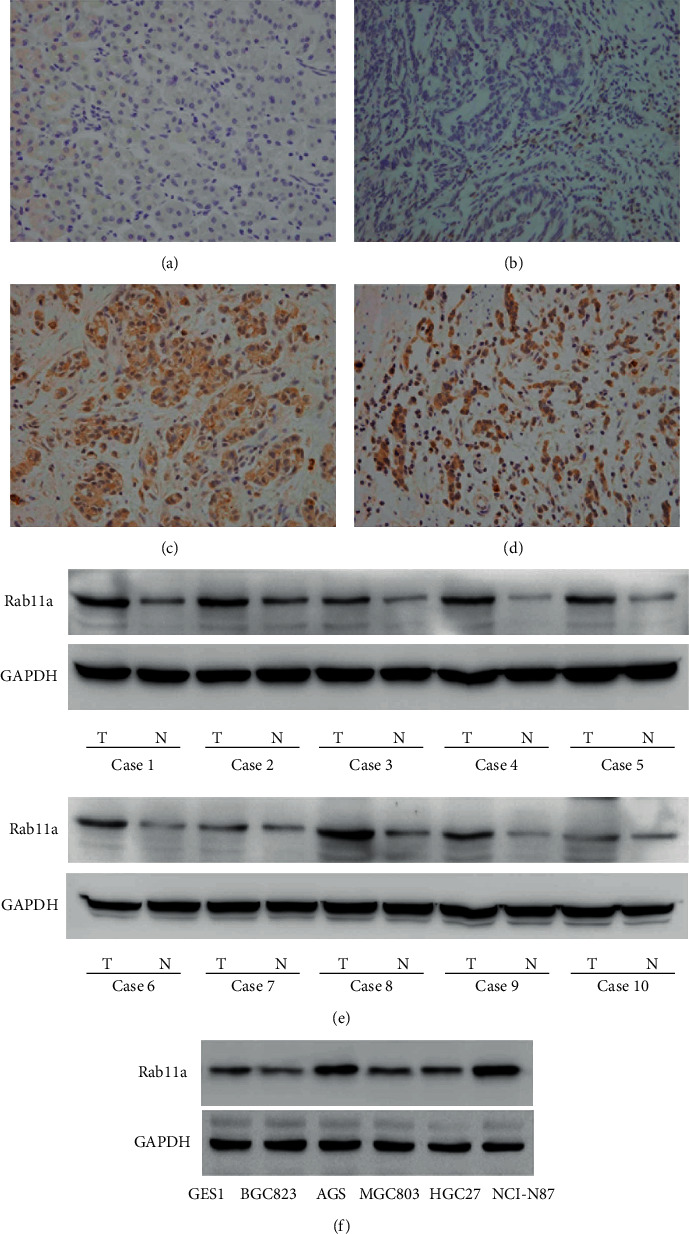
Rab11a expression is increased in gastric cancer tissues. (a) Negative immunostaining of Rab11a in a case of normal tissue. (b) Negative Rab11a immunostaining in a case of tubular adenocarcinoma. (c) Positive cytoplasmic immunostaining in a case of tubular adenocarcinoma. (d) Positive cytoplasmic Rab11a immunostaining in a case of mucinous adenocarcinoma. (e) Western blotting of Rab11a in ten cases of cancer tissues and their adjacent normal gastric tissues. (f) Western blotting of Rab11a in GES-1 normal cell line and five cancer cell lines.

We first analyzed expression pattern of Rab11a in 108 cases of paraffin embedded gastric cancer tissues and 10 cases of normal gastric tissues using immunohistochemistry. Weak/negative staining of Rab11a was found in normal tissues (Figure 1(a)) while increased Rab11a staining was found in the cytoplasm of gastric cancer tissues. Rab11a protein expression was increased in 49 of 108 (45.3%) cases (Figures 1(b)–1(d)). The correlation of Rab11a with clinicopathological factors was shown in Table 1. Rab11a high expression was significantly associated with advanced TNM stage (*P*=0.0007), nodal metastasis (*P*=0.1115), and local invasion (T stage) (*P*=0.0011). There was no difference between Rab11a status and age, gender and tumor differentiation. Rab11a protein expression was also examined in 10 pairs of fresh gastric cancer tissues with their adjacent normal tissues using western blotting. Rab11a protein expression was obviously higher in 7 of 10 cancer tissues compared with their corresponding normal tissues (Figure 1(e)). In addition, we profiled Rab11a protein in a panel of gastric cancer cell lines and normal GES-1 cell line (Figure 1(f)). The AGS and NCI–N87 cell lines had relatively high Rab11a expression compared with GES-1 cell line.

### 3.2. Rab11a Promotes Cell Proliferation and Invasion

**Figure 2:**
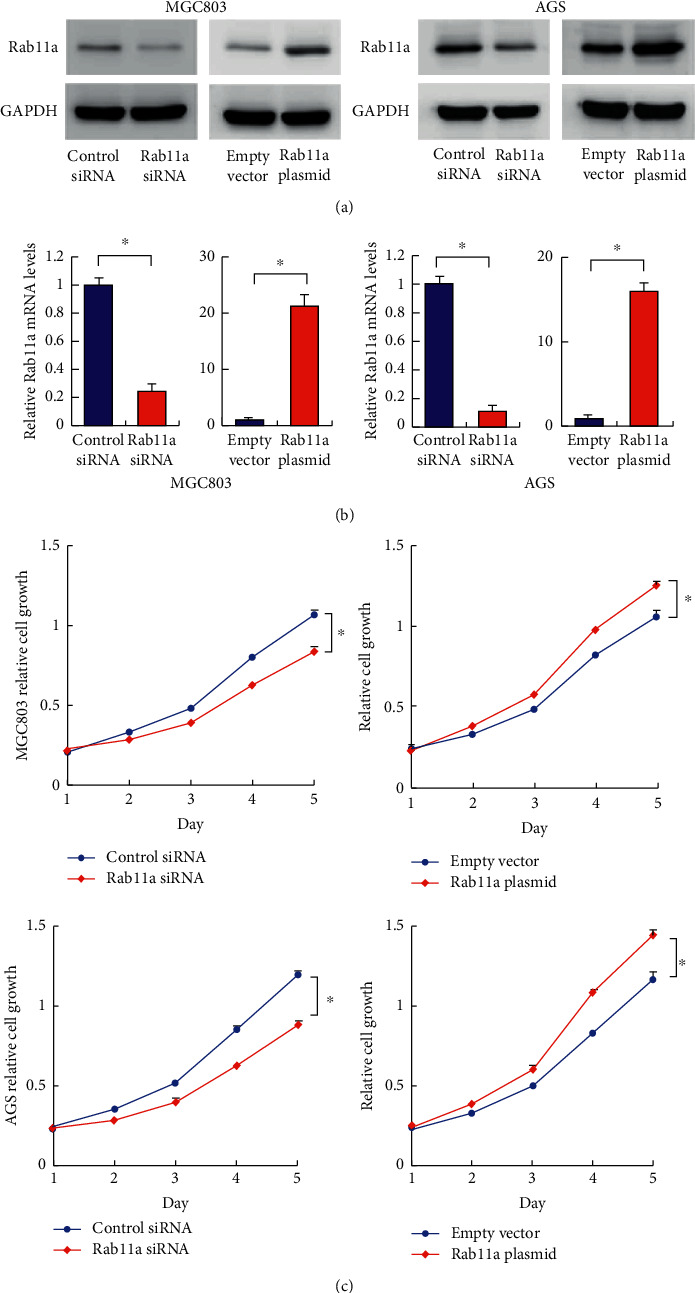
Rab11a regulates gastric cancer cell proliferation. (a) Plasmid transfection significantly upregulated Rab11a protein, while siRNA treatment downregulated Rab11a protein expression in both MGC803 and AGS cell lines. (b) Plasmid transfection significantly upregulated, while siRNA treatment downregulated Rab11a mRNA expression in both MGC803 and AGS cell lines. (c) MTT assay showed that Rab11a overexpression promoted proliferation rate, while Rab11a depletion inhibited proliferation rate in both MGC803 and AGS cell lines. ^*∗*^*P* < 0.05.

**Figure 3:**
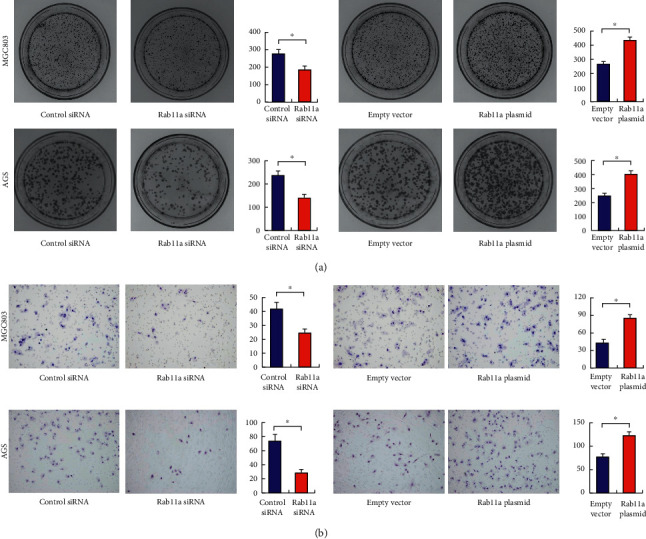
Rab11a regulates colony formation and invasion. (a) Colony formation assay showed that Rab11a overexpression upregulated colony number, while Rab11a depletion decreased colony number in both MGC803 and AGS cell lines. (b) Matrigel invasion assay showed that Rab11a overexpression increased invading cell number, while Rab11a depletion decreased invading cell number. ^*∗*^*P* < 0.05.

Rab11a overexpression and siRNA knockdown were performed in both MGC803 and AGS cell lines. The transfection efficiency was confirmed by RT-qPCR and western blot (Figures 2(a) and 2(b)). MTT assay showed that Rab11a overexpression increased proliferation rate, while Rab11a siRNA decreased proliferation rate in both MGC803 and AGS cells (Figure 2(c)). Colony formation assay showed that Rab11a overexpression increased regulated colony formation numbers, while Rab11a depletion decreased colony numbers (Figure 3(a)). In addition, Matrigel invasion assay showed that Rab11a overexpression increased the invading cell numbers, while Rab11a knockdown decreased invading ability in both MGC803 and AGS cell lines.

### 3.3. Rab11a Downregulates Cisplatin Sensitivity

**Figure 4:**
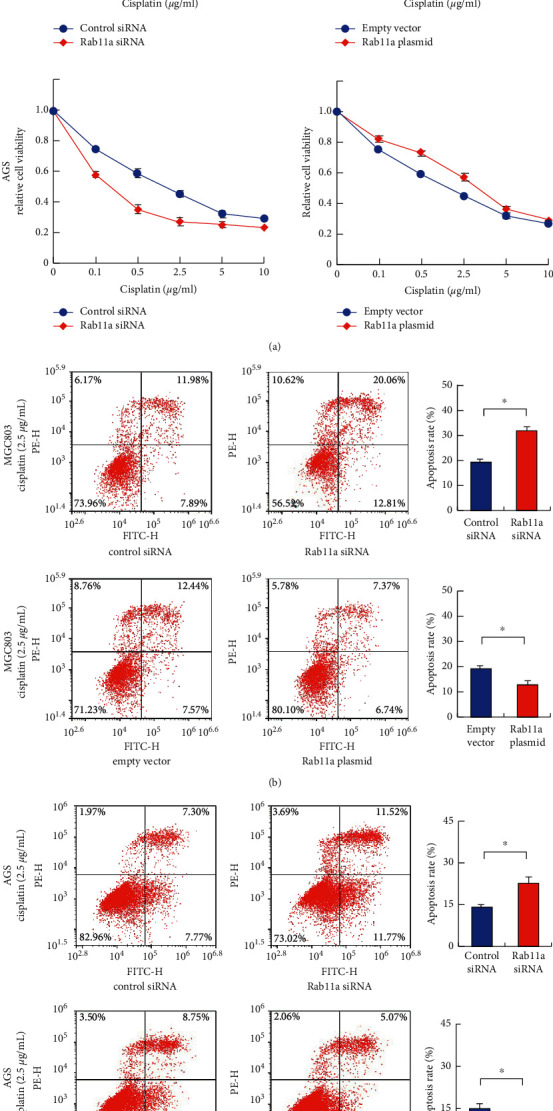
Rab11a regulates cisplatin sensitivity. (a) MTT demonstrated that Rab11a overexpression decreased inhibition rate, while Rab11a depletion increased inhibition rate in gastric cancer cells treated with different concentration of cisplatin. (b) Annexin V/PI analysis showed that Rab11a overexpression decreased apoptosis, while (c) Rab11a depletion increased apoptosis rate in gastric cancer cells treated with cisplatin. ^*∗*^*P* < 0.05.

Next, we investigated the effect of Rab11a on cisplatin sensitivity. We used different concentration of cisplatin (0.1, 0.5, 2.5, 5, and 10 *μ*g/mL) to treat gastric cancer cells with Rab11a overexpression and depletion and checked the inhibition rate of cisplatin on cell viability using MTT assays. Rab11a overexpression decreased inhibition rate, while Rab11a depletion increased inhibition rate (Figure 4(a)). The apoptosis rate was examined using Annexin V/PI staining. As shown in Figures 4(b) and 4(c), Rab11a overexpression reduced levels of cisplatin-induced apoptosis (2.5 *μ*g/mL, 24 h), while Rab11a knockdown increased cisplatin-induced apoptosis in both MGC803 and AGS cell lines, indicating Rab11a conferred resistance to cisplatin treatment.

### 3.4. Rab11a Maintains Mitochondrial Membrane Potential

**Figure 5:**
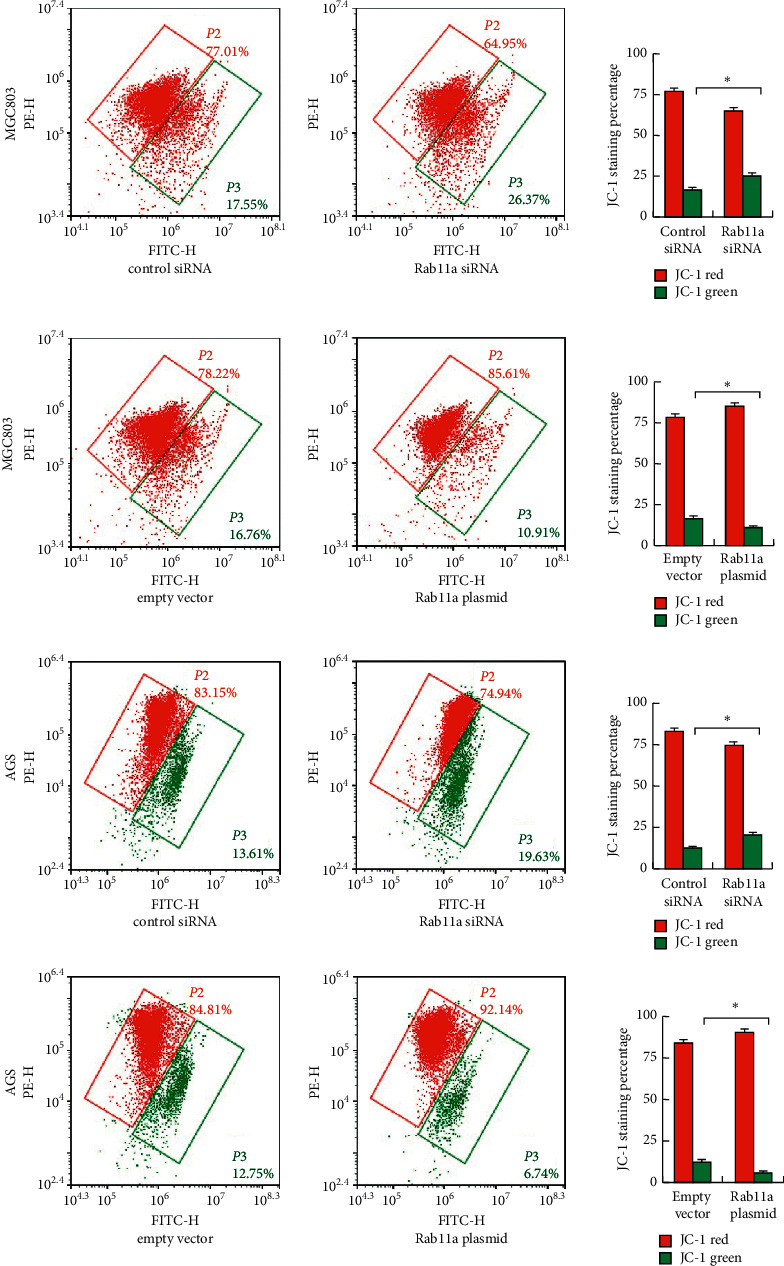
Rab11a regulates mitochondrial membrane potential. JC-1 staining was used to stain MGC803 and AGS cells treated with cisplatin. Flow cytometry showed that Rab11a overexpression increased mitochondrial membrane potential with increased JC-1 red/green ratio, while Rab11a depletion decreased mitochondrial membrane potential with decreased JC-1 red/green ratio in both MGC803 and AGS cell lines. ^*∗*^*P* < 0.05.

Maintaining normal mitochondrial membrane potential (Δ*ψ*m) is important during cell survival when treated with chemotherapeutic drugs. We examined whether Rab11a regulates Δ*ψ*m using JC-1 staining. JC-1 staining exhibits intense red fluorescence, while it turns green when Δ*ψ*m is decreased. As shown in Figures 5(a) and 5(b), Rab11a reduced the percentage of green staining, while Rab11a depletion increased green staining percentage in both MGC803 and AGS cells treated with cisplatin. These findings suggested Rab11a could maintain normal Δ*ψ*m in gastric cancer cells during cisplatin treatment.

### 3.5. Rab11a Regulates Bcl-2, MMP2, Cyclin D1, p-FAK, and p-AKT

**Figure 6:**
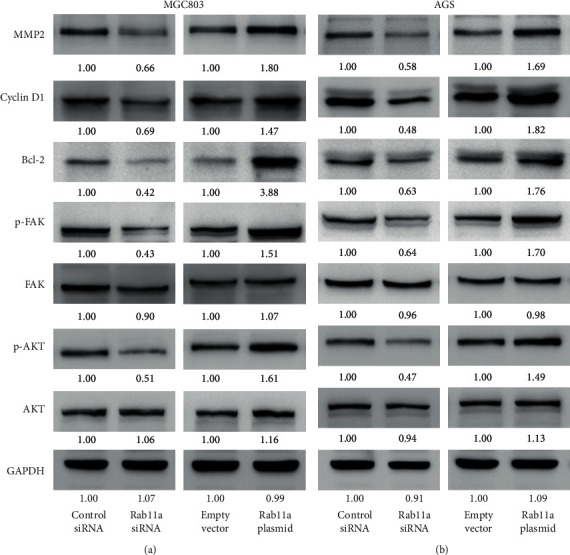
Rab11a regulates MMP2, cyclin D1, Bcl-2, p-FAK, and p-AKT. Western blot showed that Rab11a overexpression upregulated the protein expression of MMP2, cyclin D1, and Bcl-2. Rab11a also increased phosphorylation of FAK and AKT. Rab11a siRNA showed the opposite effects on these proteins. Quantification of western blot that was performed was indicated.

To further elucidate the underlying mechanisms of Rab11a, we checked changes of several related proteins and signaling pathways. As shown in Figure 6, Rab11a overexpression upregulated MMP2, cyclin D1, and Bcl-2 protein levels. Rab11a overexpression also increased phosphorylation of FAK and AKT. Rab11a depletion decreased protein levels of Bcl-2, MMP2, cyclin D1, p-FAK, and p-AKT in both MGC803 and AGS cell lines.

### 3.6. Rab11a Regulates Cyclin D1, MMP2, and Bcl-2 through FAK/AKT Signaling Pathway

**Figure 7:**
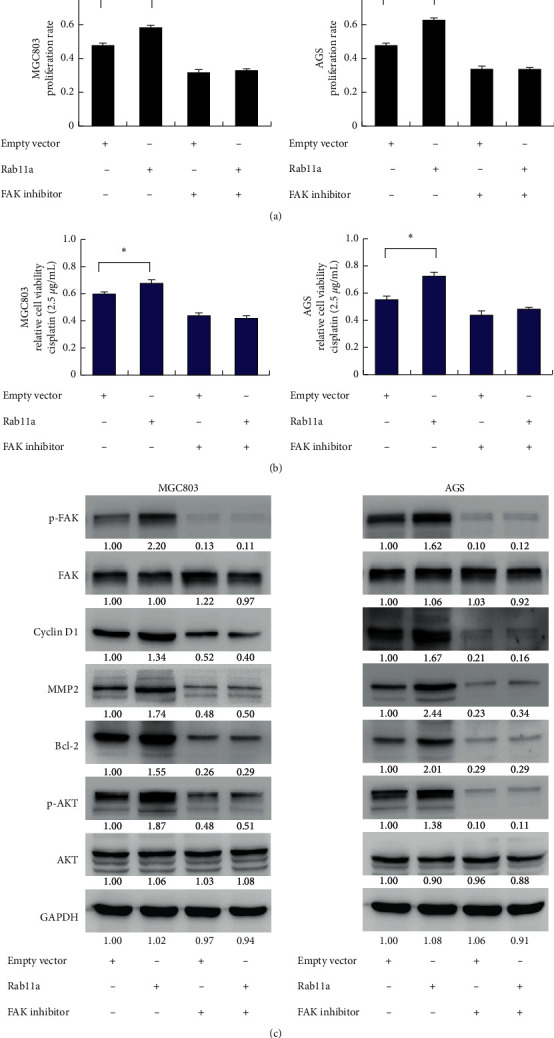
Rab11a regulates proliferation, chemosensitivity and related proteins through FAK signaling. (a) MTT assay showed that FAK inhibitor downregulated cell viability 3 days after Rab11a transfection. FAK inhibitor also abolished the growth promoting effect of Rab11a. (b) Cell viability was examined after 2.5 *μ*g/mL cisplatin treatment. FAK inhibitor suppressed Rab11a mediated cisplatin resistance. (c) FAK inhibitor PF-573228 was used in cells transfected with Rab11a plasmid or control vector. PF-573228 significantly decreased phosphorylation of AKT and FAK. PF-573228 also decreased cyclin D1, MMP2, and Bcl-2 protein expression. In cells treated with PF-573228, Rab11a did not increase cyclin D1, MMP2, and Bcl-2 protein. Quantification of western blot that was performed was indicated.

Activation of FAK signaling has been reported to induce cyclin D1, MMP2, and Bcl-2 expression. FAK also acts upstream of AKT signaling. To validate the interaction between FAK signaling and Rab11a, we used FAK inhibitor PF-573228 in cells transfected with Rab11a plasmid. MTT assay showed that FAK inhibitor abolished the growth promoting effect of Rab11a (Figure 7(a)). We also tested cell viability after 2.5 *μ*g/mL cisplatin treatment. As shown in Figure 7(b), FAK inhibitor suppressed Rab11a mediated cisplatin resistance. In addition, FAK inhibitor significantly downregulated its phosphorylation level. Treatment with FAK inhibitor decreased AKT phosphorylation, cyclin D1, Bcl-2, and MMP2 (Figure 7(c)). There was significant amelioration of Rab11a-induced upregulation of cyclin D1, Bcl-2, and MMP2, implying that effects of Rab11a upon these protein were dependent upon FAK signaling. These data suggested that FAK played a central role during Rab11a-induced increase of cyclin D1, Bcl-2, and MMP2 in gastric cancer cells.

## 4. Discussion

Recent evidences suggested Rab11a as a cancer-related protein. Rab11a overexpression has been reported in non-small-cell lung cancer, pancreatic cancer, and colorectal carcinoma [6–8]. To date, its expression pattern in gastric cancers remains unclear. Our current study demonstrated that Rab11a protein was elevated in gastric cancers and correlated with nodal metastasis, higher TNM stage, and local invasion. To explore its biological roles, we picked MGC803 and AGS cell lines for further study. Our results showed that Rab11a promoted cancer cell growth. Accordingly, Rab11a could positively regulate cyclin D1 protein, suggesting Rab11a accelerated gastric cancer growth through induction of cell cycle regulators.

Cancer invasion is a multiple step process which includes proteolytic remodeling of the basement membrane and extracellular matrix (ECM). Our study found that Rab11a increased invasion ability and positively regulated MMP2, a potent protease that could degrade ECM and promotes tumor cell invasion, suggesting Rab11a promotes invasion through MMP2.

Cisplatin is a first-line chemotherapeutic drug which triggers apoptosis. During clinical practice, gastric cancers often develop cisplatin resistance which greatly limited its efficiency. Thus, it is important to investigate mechanism of regulation of cisplatin sensitivity. The role and mechanism of Rab11a on cisplatin sensitivity has not been previously reported. We found that Rab11a overexpression decreased cisplatin sensitivity and reduced cisplatin-induced apoptosis in gastric cancer cells. Mitochondrial status plays an important role during cisplatin induce apoptosis [10–12]. Cisplatin treatment results in loss of mitochondrial membrane potential, which increases mitochondrial permeability and triggers cytochrome c release into cytosol and activates apoptosis pathways. Our data showed that Rab11a overexpression could maintain mitochondrial membrane potential during cisplatin treatment, suggesting Rab11a might inhibit apoptosis and reduce cisplatin sensitivity through protection of mitochondrial function. Accordingly, western blot showed that Rab11a upregulated Bcl-2 protein expression. Bcl-2 has been reported as a cytoprotective protein that prevents mitochondrial permeability transition pore opening and release of apoptogenic proteins from mitochondria, thus blocking loss of mitochondrial membrane potential [13]. It is possible that Rab11a prevented loss of Δ*ψ*m through Bcl-2 upregulation during cisplatin treatment.

To search for the possible mechanism of Rab11a on cyclin D1, MMP2, and Bcl-2 upregulation, we screened several signaling pathways and our results showed that Rab11a upregulated both FAK and AKT signaling. Previous reports showed that FAK signaling was activated in various human cancers including gastric cancer [14, 15]. Activation of FAK correlated with accelerated cancer growth and invasion [16]. FAK contributes to cell cycle progression by transcriptional activation of cyclin D1 promoter [17]. FAK has also been reported to regulate expression of MMP-2 [18], and its inhibitor reduced MMP-2 and invasion in cancer cells [19, 20]. In addition, FAK could activate downstream AKT/Bcl-2 signaling, which reduces apoptosis [20, 21]. To validate their relationship in gastric cancer cells, we used FAK inhibitor to block its function and the effects of Rab11a on cyclin D1, MMP2, and Bcl-2 were significantly reduced. FAK inhibitor also abolished Rab11 mediated proliferation and cisplatin resistance. These findings suggest a link between Rab11a, FAK, and gastric cancer growth/chemo-resistance. There is a link between the function of Rab11a in GC and in other cancers. Rab11a promotes proliferation and invasion in lung cancer and pancreatic cancer [7, 8]. Rab11 upregulates MMP2 and activates AKT signaling in hepatocellular carcinoma [22], suggesting the function of Rab11a might share some similarities.

In conclusion, the current study identified novel roles of Rab11a as oncoprotein overexpressed in human gastric cancers. Rab11a promoted gastric cancer proliferation, invasion, and cisplatin resistance and prevented loss of mitochondrial membrane potential possibly through FAK/AKT signaling. Our findings provided the possibility of targeting Rab11a-FAK/AKT axis as a potential therapeutic strategy.

## Figures and Tables

**Table 1:** Correlation of Rab11a expression with clinicopathological characteristics in gastric cancer.

Characteristics	Numbers	Rab11a low expression	Rab11a high expression	*P*
Gender				
Male	83	45	38	0.8753
Female	25	14	11	

Age				
<60	57	34	23	0.2680
≥60	51	25	26	

Differentiation				
Poor–moderate	61	35	26	0.5135
Well	47	24	23	

Tumor invasion (T)				
T1 + T2	40	30	10	0.0011
T3 + T4	68	29	39	

Lymph node metastasis				
Absent	38	27	11	0.0115
Present	70	32	38	

TNM stage				
I	21	19	2	0.0007
II	30	16	14	
III	57	24	33	

## Data Availability

The data that support the findings of this study are available on request to the corresponding author.
